# High in‐vivo accuracy of a novel robotic‐arm‐assisted system for total knee arthroplasty

**DOI:** 10.1002/ksa.12272

**Published:** 2024-05-20

**Authors:** Faseeh Zaidi, Craig M. Goplen, Connor Fitz‐Gerald, Scott M. Bolam, Michael Hanlon, Jacob T. Munro, Andrew P. Monk

**Affiliations:** ^1^ Department of Surgery University of Auckland Auckland New Zealand; ^2^ Auckland Bioengineering Institute University of Auckland Auckland New Zealand; ^3^ Department of Orthopaedic Surgery Auckland City Hospital Auckland New Zealand; ^4^ Department of Surgery University of Alberta Edmonton Alberta Canada

**Keywords:** accuracy, robotic surgery, ROSA total knee system, total knee arthroplasty

## Abstract

**Purpose:**

Robotic‐assisted total knee arthroplasty (TKA) has been shown to improve the accuracy and precision of bony resections and implant position. However, the in vivo accuracy of the full surgical workflow has not been widely reported. The primary objective of this study is to determine the accuracy and precision of a robotic‐arm‐assisted system throughout the intraoperative workflow.

**Methods:**

This was a retrospective cohort study of adult patients who underwent primary TKA with various workflows and alignment targets by three arthroplasty‐trained surgeons with previous experience using the ROSA® Knee System (Zimmer Biomet) over a 3‐month follow‐up period. Accuracy and precision were determined by measuring the difference between various workflow time points, including the final preoperative plan (PP), robot‐validated (RV) resection angle and postoperative radiographs (PR). The absolute mean difference between the measurements determined accuracy, and the standard deviation represented precision. The lateral distal femoral angle, medial proximal tibial angle, femoral flexion angle and tibial slope were measured on postoperative coronal long‐leg radiographs and true short‐leg lateral radiographs.

**Results:**

A total of 77 patients were included in the final analyses. The accuracy for the coronal femoral angle was 1.62 ± 1.11°, 0.75 ± 0.79° and 1.96 ± 1.29° for the differences between PP and PR, PP and RV and RV and PR. The tibial coronal accuracy was 1.44 ± 1.03°, 0.81 ± 0.67° and 1.57 ± 1.14° for PP/PR, PP/RV and RV/PR, respectively. Femoral flexion accuracy was 1.39 ± 1.05°, 0.83 ± 0.59° and 1.81 ± 1.21° for PP/PR, PP/RV and RV/PR, respectively. Tibial slope accuracy was 0.99 ± 0.72°, 1.19 ± 0.87° and 1.63 ± 1.11°, respectively. The proportion of patients within 3° was 93.2%, 95.3%, 97.3% and 94.6% for the distal femur, proximal tibia, femoral flexion and tibial slope angles when the final intraoperative plan was compared to PRs. No patients had a postoperative complication at the final follow‐up.

**Conclusions:**

The ROSA Knee System has acceptable accuracy and precision of coronal and sagittal plane resections with few outliers at various steps throughout the platform's entire workflow in vivo.

**Level of Evidence:**

Level III.

**Abbreviations**
AHREC: Auckland Health Research Ethics Committee
ASA: American Society of Anaesthesiologists
BMI: body mass index
CT: computed tomography
HKA: hip–knee–ankle
mLDFA: mechanical lateral distal femoral angle
mMPTA: mechanical medial proximal tibial angle
MRI: magnetic resonance imaging
PP: preoperative plan
PR: postoperative radiographs
RA‐TKA: robotic‐assisted total knee arthroplasty
RV: robot‐validated
SD: standard deviation
TKA: total knee arthroplasty

## INTRODUCTION

Recent technological advancements have led to the introduction of robotic‐assisted total knee arthroplasty (RA‐TKA) to improve the accuracy and precision of bony resections and implant position [12, 20]. Early RA‐TKA platforms were criticised due to increased procedural complexity, cumbersome interfaces requiring specialised medical personnel and additional expenses such as preoperative computed tomography (CT) scans [20]. Current RA‐TKA platforms offer various features: semiactive haptic platforms enable the robot to generate the force required to perform the necessary cut with direct auditory, tactile or visual feedback to the surgeon, while other passive systems utilise an articulating arm under direct, continuous surgeon control with instruments recognised by a navigation system [12]. In contrast, active platforms do not rely on any surgeon input during the placement and execution of the end effector [14].

A novel collaborative computer‐based platform can utilise a robotic arm to place a cutting block based on an operative plan obtained using predefined anatomic landmarks [9]. This enables surgeons to precisely perform the femoral and tibial resections independently with a conventional oscillating saw [9]. These platforms can be supplemented with preoperative radiographs to create patient‐specific three‐dimensional (3D) models compatible with various TKA alignment philosophies and workflows, offering registration accuracy rivalling magnetic resonance imaging (MRI) [10].

Initial cadaveric studies report high precision and accuracy with this platform [13, 18]. While several reports [8, 9, 16, 17] demonstrate highly accurate alignment parameters measured via postoperative radiographs among a cohort of patients undergoing RA‐TKA with this system, Shin et al. reported variability in postoperative sagittal alignment accuracy with the same platform [19]. Nevertheless, calls have been made to expand the in vivo evidence of RA‐TKA [3, 5].

An important step within the RA‐TKA intraoperative workflow that may influence accuracy outcomes is the ability to accurately register anatomic landmarks. Charette et al. evaluated the reliability of landmark registration on two cadaveric knee specimens between three surgeons and reported intrarater intra‐class correlation coefficients (ICCs) of 0.767–0.988 and inter‐rater ICCs of 0.89–0.997 [4]. While these results suggest excellent landmark registration reliability, it is important to note that differences may exist between cadaveric and in vivo landmarking. For example, the potential for errors in the sagittal alignment exists across all major robotic platforms [1]. For the platform studied herein, this error potential is a result of inconsistency when choosing the tibial canal entry point during landmarking. Most in vivo studies have investigated alignment differences between the intraoperative plan and the robot‐validated measures [7, 16, 19] or the intraoperative plan postoperative radiographs [7, 9, 16, 19]. However, sometimes secondary cuts or soft tissue releases are necessary after reviewing the intraoperative robot‐validated measures. Inaccuracies in the validation step may, therefore, affect final component positioning. As such, it is important to assess the accuracy and precision across the entire workflow, comparing the intraoperative plan to the robot‐validated measures, the robot‐validated measures to the postoperative radiographic alignment and the intraoperative plan to the postoperative radiographic alignment. To our knowledge, only one study has reported on all three comparisons. Mancino et al. reported differences of less than 1.0° between all measures taken at each step in the workflow for the tibial and femoral coronal and sagittal angles [9]. In this study, the error between intraoperative planned and robotic‐validated for the femoral flexion and the tibial coronal angle was significantly greater than the error found between intraoperatively planned and postoperatively measured or robotic‐validated and postoperatively measured. Given the lack of studies examining accuracy and precision throughout the workflow, and the inconsistencies reported by Mancino et al., a direct comparison of in vivo postoperative component and limb alignment with intraoperative measurements using the robotic system is required.

The primary objective of this study was to investigate the accuracy and precision of a cut‐block positioning robotic arm by comparing intraoperative implant positioning and limb alignment with postoperative radiographic component alignment in primary RA‐TKA.

## METHODS

### Study design

This was a retrospective cohort study of prospectively collected data on patients with primary knee osteoarthritis undergoing primary, unilateral RA‐TKA using the ROSA® Knee System (Zimmer Biomet). Ethics approval was obtained from the local and institutional ethical committee prior to the commencement of the study (AHREC: 000072). Adults aged ≥50 years who underwent primary knee arthroplasty for symptomatic end‐stage knee osteoarthritis between February 2020 and July 2022 with a 3‐month follow‐up were included. Exclusion criteria were previous knee surgery with implants in or around the joint, revision from unicompartmental knee arthroplasty to TKA, infection, neurological dysfunction limiting knee mobility or posttraumatic osteoarthritis with severe knee deformity (defined as a varus or valgus angle ≥10°). Three fellowship‐trained arthroplasty surgeons performed all RA‐TKA at a single centre (Auckland City Hospital, Auckland, New Zealand) who had previously trained on the robotic system with a minimum of 10 RA‐TKAs in Auckland, New Zealand [2].

### Operative technique

All patients had standardised preoperative standing long‐leg hip–knee–ankle radiographs and short‐leg lateral radiographs and surgical workflow was based on surgeon preference using measured resection or gap‐balancing techniques as previously described by this group [2].

A standard midline incision and medial parapatellar approach were used to perform an arthrotomy. After osteophytes were removed, 3.2 mm calibration pins were placed in the distal femur and proximal tibia within the surgical incision. Standard inputs included femoral and tibial anatomic registration landmarks and a dynamic soft tissue examination performed by moving the knee from full extension to full flexion with both a varus and valgus stress [4]. Planned patient alignment parameters and bony resection were finalised after flexion and extension gaps were balanced using a validated computer‐assisted navigation system. Surgeons used a personalised alignment philosophy; a neutral mechanical axis was not always the target. Personalised workflows included either ligament‐referenced resections using the Zimmer Fuzion® device (Zimmer Biomet) or measured resection with either anterior or posterior referencing.

### Outcome measures

**Figure 1:**
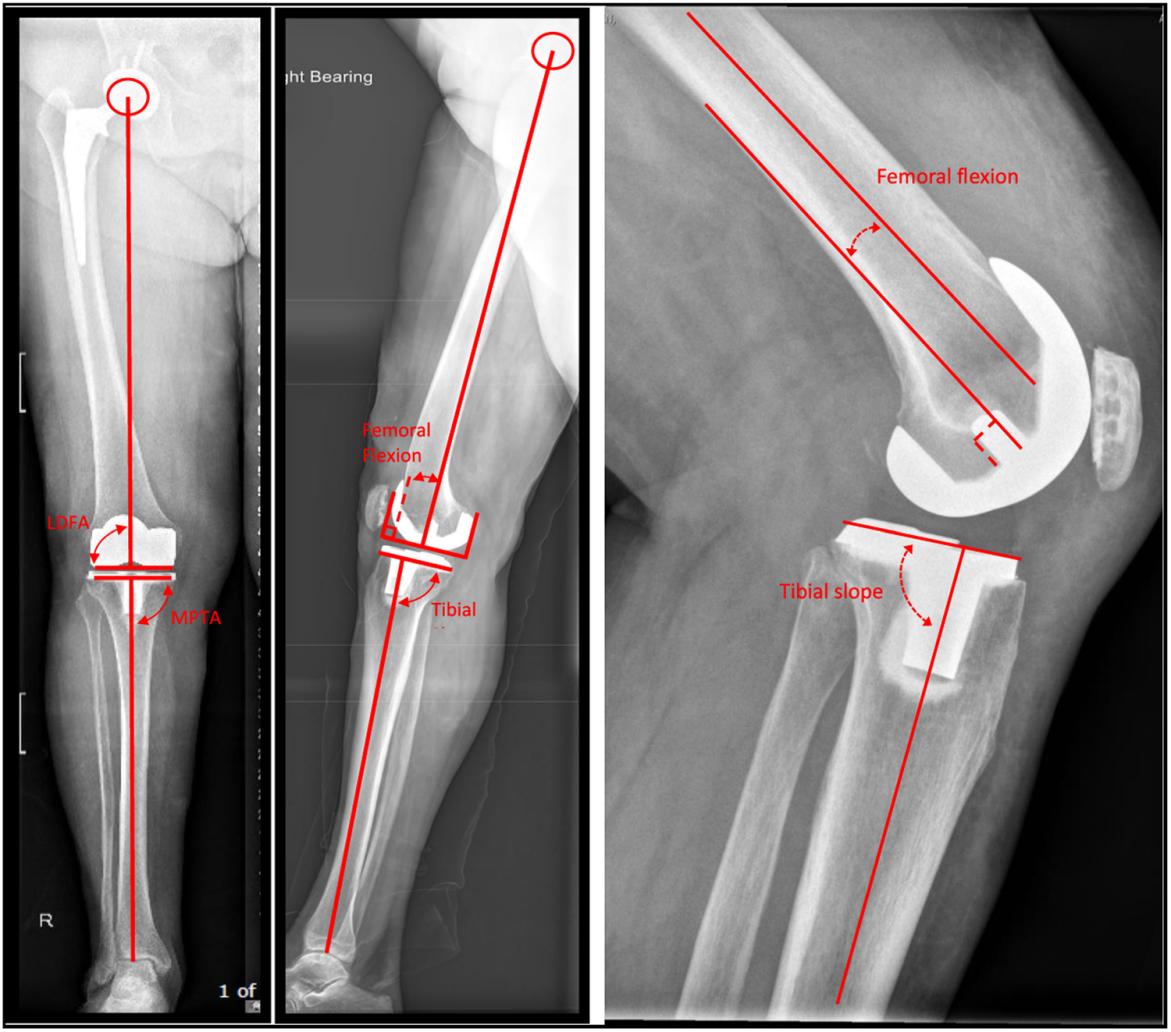
Measurement of postoperative long‐leg coronal and sagittal and short‐leg sagittal radiographs as previously described [18]. Coronal plane alignment (lateral distal femoral angle [LDFA] and mechanical medial proximal tibial angle [MPTA]) was evaluated on long‐leg anteroposterior radiographs. Sagittal plane alignment (femoral flexion and tibial slope) was evaluated on short‐leg and long‐leg lateral radiographs.

The primary outcome measure was the difference between the final recorded intraoperative plan and postoperative radiographic coronal and sagittal measurements. Our secondary outcome measures were the difference between (a) the final intraoperative planned resection angles and the validated resection angle (cutting error) and (b) between the validated resection angle and postoperative radiographic coronal and sagittal measurements. Validated resection angles were determined after final cuts were performed and verified using a ROSA validation device. The mechanical lateral distal femoral angle (LDFA), mechanical medial proximal tibial angle (MPTA), femoral flexion angle and posterior tibial slope were measured on postoperative coronal long‐leg radiographs and true short‐leg lateral radiographs as described previously (Figure 1) [11]. Coronal alignment was evaluated on the long‐leg anteroposterior radiographs. LDFA was measured as the lateral angle between the distal femoral component surface with respect to the mechanical axis of the femur. MPTA was measured as the medial angle between the baseplate and the mechanical axis of the tibia. Sagittal alignment was evaluated on short‐leg or long‐leg lateral radiographs. Femoral flexion was measured as the angle between the most distal femoral fixation surface with respect to the femoral shaft axis. The tibial slope was measured as the angle formed between the anatomical axis of the tibia and the baseplate tangent. Postoperative complications such as those directly related to the use of the ROSA platform including wound complications associated with pin sites, periprosthetic fractures or fractures at pin sites were noted.

Accuracy was the mean difference between measurements, while the standard deviation represented precision [6]. Outliers were defined as the final component position being >±3° compared to the intraoperative validation plan. Each observer was blinded to the other's measures.

Two investigators (F. Z. and C. M. G) independently performed all the radiological measurements, with each observer blinded to the other's measurements. The final angles were based on the average of the two measurements. A third independent reviewer was used to assess the radiograph if a disagreement between measured angles was more than 3°. The inter‐rater agreements for each mechanical alignment measurement were evaluated using a Bland–Altman plot (Appendix A). Each demonstrated good agreement around the zero bias line and within the 95% confidence interval.

In addition, nine ‘outlier’ (>±3° between inter‐rater measurements after measuring from true short‐leg lateral radiographs) patients within the cohort were brought back to obtain long‐leg lateral radiographs to remeasure the sagittal plane parameters. The values reported for tibial slope and femoral flexion angle reflect the average mean and standard deviation of these nine patients.

### Statistical analysis

All statistical analyses and graphing were performed with PRISM 8 (GraphPad version 10.2.2). A *p* < 0.05 was considered statistically significant. Continuous data are presented by means ± standard deviations (SDs) with 95% confidence intervals, while counts and percentages are used for categorical data. The difference between the final intraoperative planned angle and robot‐validated resection angle and postoperative imaging angles are reported as absolute values. Differences between the planned resection angle, ROSA‐validated angle and radiographic alignment were compared with a one‐way analysis of variance (ANOVA). Mean absolute errors (MAEs) between the planned resection angle versus ROSA‐validated angle, ROSA‐validated angle versus postoperative radiographic alignment and planned resection angle versus postoperative radiographic alignment were compared with a one‐way ANOVA.

## RESULTS

**Figure 2:**
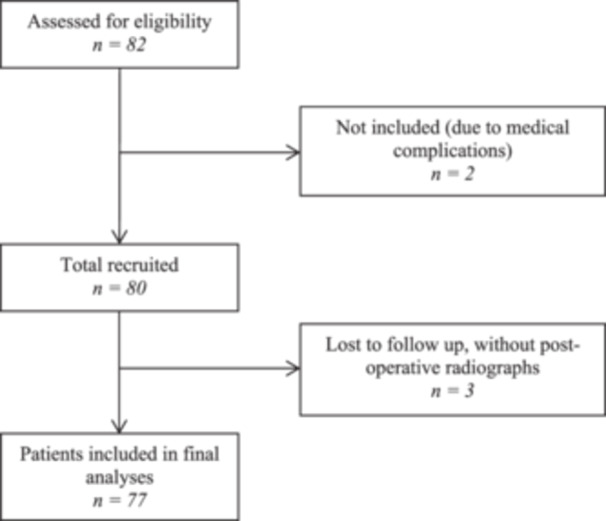
Flow diagram of patients assessed for eligibility and included in the study for final analyses.

Seventy‐seven patients met the inclusion criteria and underwent RA‐TKA. 66.2% of participants were female, and the entire cohort had a mean body mass index of 31.6 ± 5.9 kg/m² with a mean age of 69.8 ± 9.2 years (Table 1). Three patients (3.9%) were lost to follow‐up, did not complete postoperative radiographs and were excluded (Figure 2).

**Table 1:** Patient characteristics and operative details.

Age (years)^a^	69.8 (±9.2)
Gender^b^	
Male	26 (33.8%)
Female	51 (66.2%)
BMI (kg/m^2^)^a^	31.6 (±5.9)
ASA^b^	
I	6 (7.8%)
II	51 (66.2%)
III	19 (24.7%)
IV	1 (1.3%)
Operative site^b^	
Left	42 (54.5%)
Right	35 (45.5%)
Follow‐up (days)^a^	92.3 (±4.7)

Abbreviations: ASA, American Society of Anesthesiologists physical status; BMI, body mass index.

a Values reported as mean with the standard deviation in parenthesis.

^b^ Values reported as the number of patients with the percentage in parenthesis.

There were no significant (*p* > 0.05) differences between planned, validated or postoperative radiographs for any of the measured angles (Supplementary Table 1).

**Figure 3:**
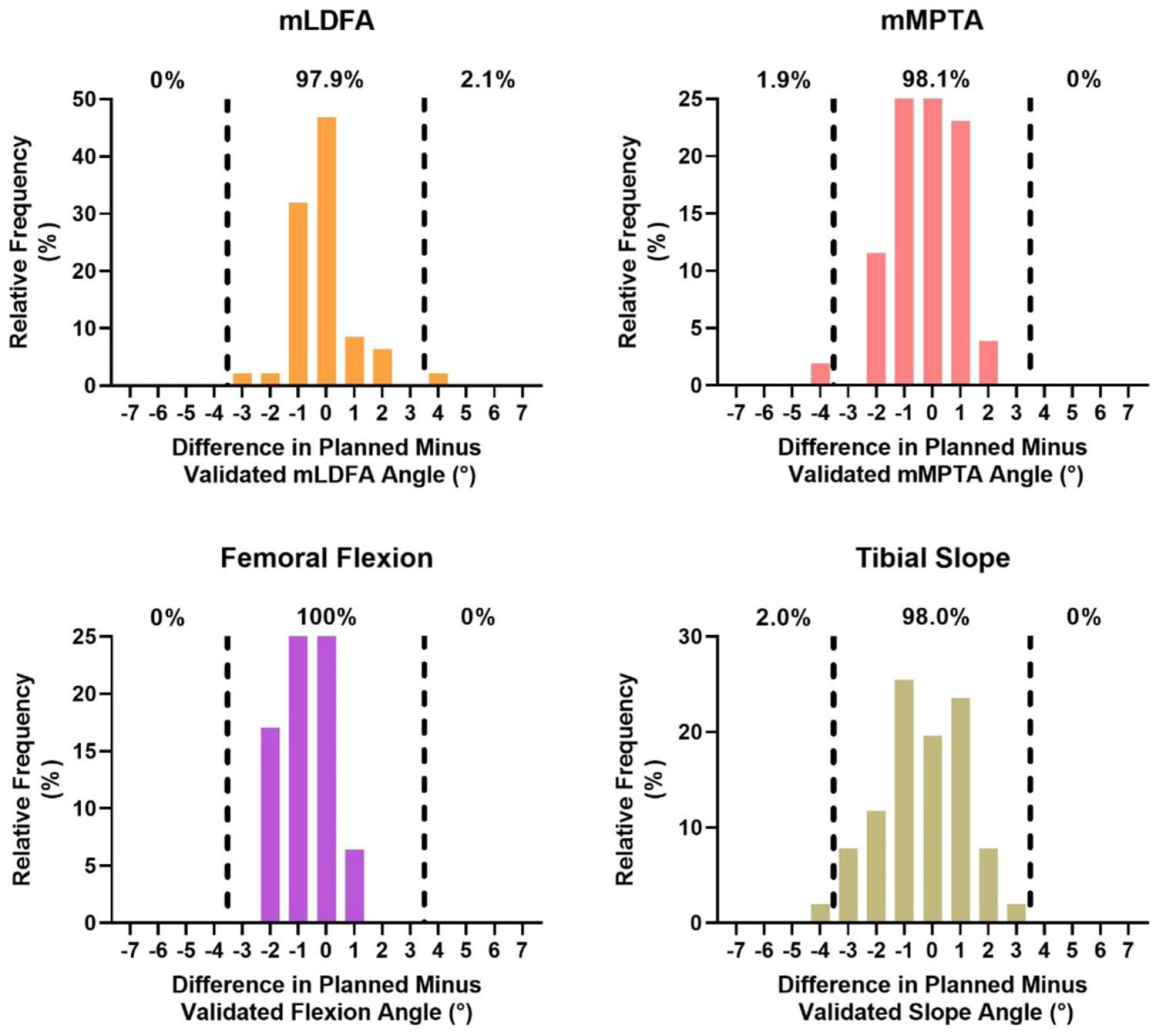
Difference between final intraoperative planned resection angle and the robotic‐validated coronal and sagittal alignment. Dotted lines indicate greater than 3° of intraoperative planned cut. mLDFA, mechanical lateral distal femoral angle; mMPTA, mechanical medial proximal tibial angle.

**Figure 4:**
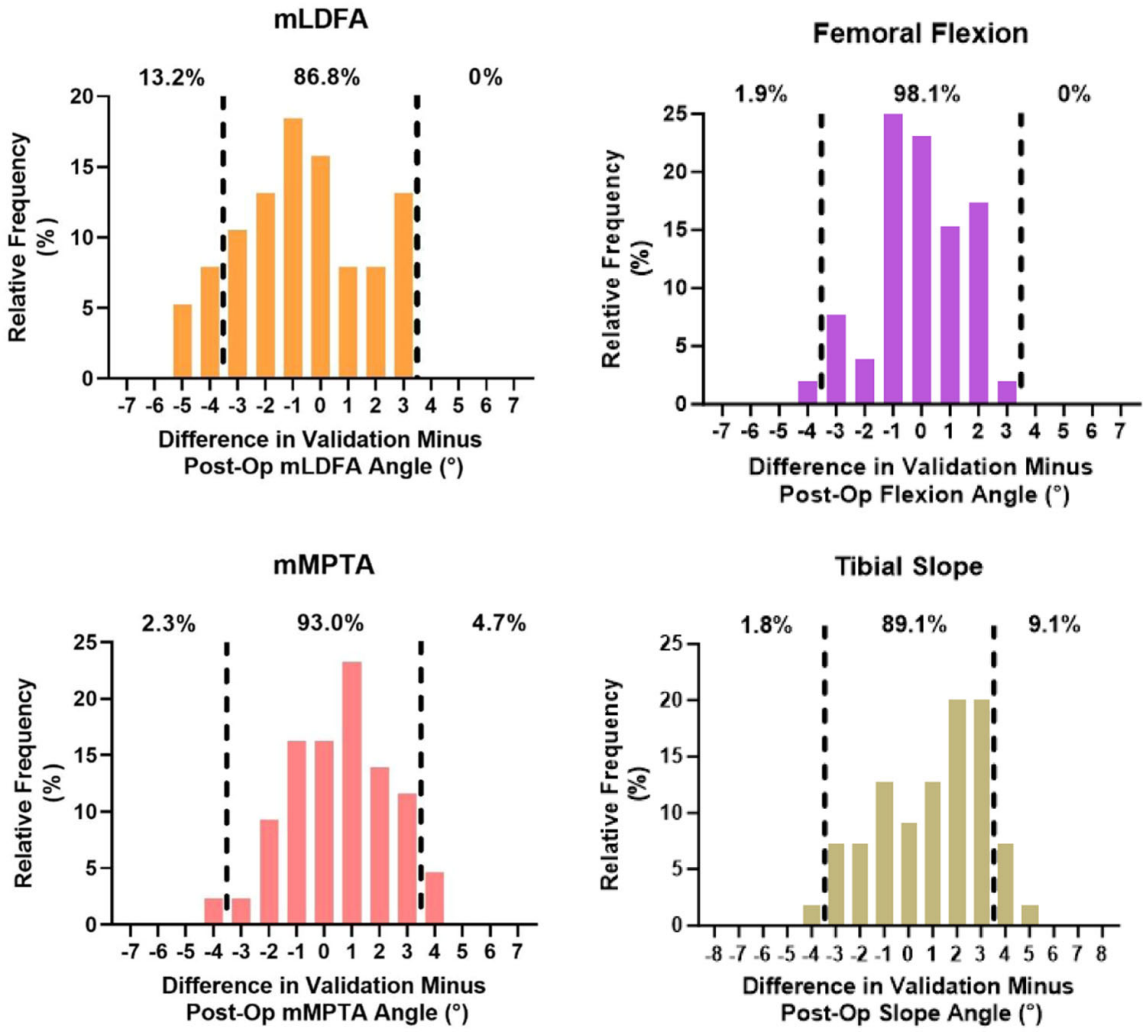
Difference between the robotic‐validated and postoperative radiographic coronal and sagittal alignment. Dotted lines indicate greater than 3° of intraoperative planned cut. mLDFA, mechanical lateral distal femoral angle; mMPTA, mechanical medial proximal tibial angle

**Figure 5:**
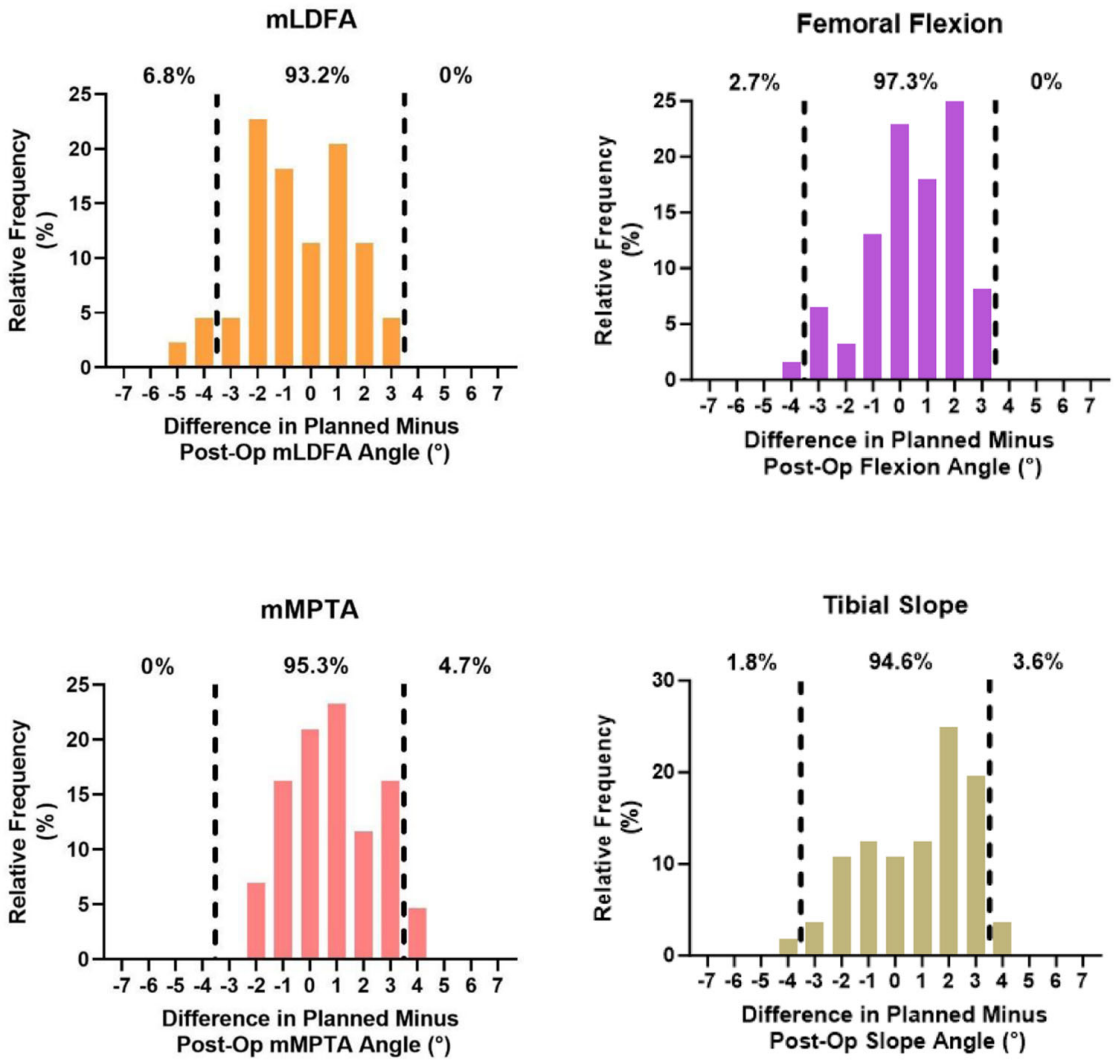
Difference between the final intraoperative planned resection angle and postoperative radiographic coronal and sagittal alignment. Dotted lines indicate greater than 3° of intraoperative planned cut. mLDFA, mechanical lateral distal femoral angle; mMPTA, mechanical medial proximal tibial angle.

The accuracy and precision for all angles comparing the final planned resection and validated angles was 0.90° ± 0.76° and the final validated resection angle and postoperative radiographs were 1.77° ± 1.23° and the final intraoperative plan and postoperative radiographs were 1.53° ± 1.07°. The MAEs between each comparison for each angle can be found in Table 2. There were no significant (*p* > 0.05) differences between any of the MAEs for their respective angles (Table 2). The proportion of planned versus validated angle errors within 3° ranged from 97.9% to 100% (Figure 3). The proportion of validated versus radiographically measured errors within 3° ranged from 86.8% to 98.1% (Figure 4). The proportion of planned versus radiographically measured errors within 3° ranged from 93.2% to 97.3% (Figure 5). Three patients (3.9%) had radiographic evidence of notching; none suffered a postoperative pin‐site fracture or wound complication at 3‐month follow‐up.

**Table 2:** Mean absolute errors for the differences between the final intraoperative planned resection angle, ROSA‐validated and postoperative radiographic coronal and sagittal alignments.

	Planned vs. validated		Validated vs. radiographic		Planned vs. radiographic		
Angle	MAE ± SD	95% CI	MAE ± SD	95% CI	MAE ± SD	95% CI	*p* Value
mLDFA	0.75 ± 0.79	0.57–0.93	1.96 ± 1.29	1.67–2.25	1.62 ± 1.11	1.37–1.87	0.33
mMPTA	0.81 ± 0.67	0.66–0.96	1.57 ± 1.14	1.32–1.83	1.44 ± 1.03	1.21–1.67	0.38
Femoral Flexion	0.83 ± 0.59	0.45–1.22	1.81 ± 1.21	1.02–2.60	1.39 ± 1.05	0.70–2.08	0.51
Tibial Slope	1.19 ± 0.87	0.62–1.76	1.63 ± 1.11	0.91–2.36	0.99 ± 0.72	0.52–1.46	0.47

Abbreviations: CI, confidence interval; MAE, mean absolute error; mLDFA, mechanical lateral distal femoral angle; mMPTA, mechanical medial proximal tibial angle; SD, standard deviation.

## DISCUSSION

The most critical findings of our study are that, on average, this robotic system was accurate within 2° and precision within 1.5° throughout the workflow in vivo. The platform was most accurate and precise when comparing the final preoperative plan with the postresection validation. In this study, overall accuracy and precision between the final operative plan and validation device were 0.90° and 0.76°; 98% of patients were within the 3° accuracy window for all coronal and sagittal measurements. These in vivo results are consistent with initial studies that relied on cadaveric specimens and used caliper measurements and this system's validation device. Parratte et al. reported that all resections differed by less than 0.7 mm and standard deviations below 1.1 mm using 30 cadaveric knees [13]. In addition, Seidenstein et al. reported all bone resection levels were below 0.7 mm, with standard deviations below 0.7 mm and final limb alignment below 1° on cadaveric knees [18].

The patients' coronal and sagittal parameters were accurate and precise within 2° when the final intraoperative planned resection angles or validation device were compared with postoperative radiographs (Table 2). This is comparable with results from Shin et al., which reported the coronal alignment resection angles were, on average, 0.88° and 1.24° different when planned resection and postoperative radiographs were compared [19]. In addition, Rossi et al. also reported no significant difference when comparing coronal parameters, as all differences were below 1 mm or 1° (SD < 1) [16]. Other studies have reported highly accurate and precise coronal and sagittal plane measurements after RA‐TKA using this system [16, 18].

Among our cohort of patients undergoing RA‐TKA, 93.2% and 95.3% of patients' coronal alignments were within a 3° accuracy target when the final preoperative plan was compared to postoperative radiographs. This is comparable to Shin et al., who determined 100% and 92% of the patient's distal femur and proximal tibial coronal resection angles within 3° among 37 patients who underwent RA‐TKA using the same system [19]. In the sagittal plane, 5.4% of patients were considered outliers when the tibial slope, measured using postoperative long‐leg radiographs, was compared to the final intraoperative plan. In contrast, Shin et al. reported that 26% had a tibial slope greater than 3° when planned resection angles were compared to postoperative radiographs using full‐length standing radiographs [19].

The reasons for these inconsistencies are unclear but could be due to variable anatomic landmark registration. Our observation supports this explanation as the planned and validated resection angles in both coronal and sagittal planes were very accurate and precise, with few outliers (Table 2). This suggests the robotic system makes precise and accurate resections according to the surgeon‐selected anatomic landmarks. However, if the surgeon's selected landmarks are inconsistent with predetermined registration points, the final implant position may be compromised when analysed on postoperative radiographs. We are unaware of any studies that have reported the accuracy and reproducibility of anatomical landmark registration in vivo. While Charette et al. concluded this system's 16 anatomic registration landmarks are consistent among different surgeons using cadaveric knees with limited arthritis, in vivo conditions are often more challenging [4]. Soft tissue envelopes are more restricted, with a great degree of bony deformity due to degenerative changes.

A strength of our study is that we did not seek to compare measurements to a target alignment or specific workflows. Our accuracy and precision results provided a real‐world report of this platform's applicability. We are only aware of one other study of comparable numbers that reported 71 robotic‐assisted knees, which did not report any coronal plane outliers >3° and were within 1.01° ± 0.08° in the coronal plane. However, this study did not report on the sagittal alignment [17].

The study had several limitations. While studies had demonstrated a good correlation between limb alignment measurements when postoperative long‐leg radiographs and CT scans were compared after TKA, rotation of a limb can change distal femur and proximal tibial coronal alignment measurements by at least 2° [10, 15]. In our study, we had a standardised preoperative and postoperative coronal long‐leg radiographic protocol to try and minimise rotational errors. However, we relied on short‐leg lateral radiographs to determine sagittal parameters, which are another potential source of error and should be investigated in the future. To overcome this, we brought back nine patients to obtain long‐leg lateral radiographs and remeasured the sagittal parameters. The results demonstrated greater accuracy and precision of the robotic system in the sagittal plane, compared to short‐leg lateral radiographic measurements. However, a conclusive statement cannot be made given the small sample size of the study cohort with long‐leg lateral radiographs. Further work is needed to establish image‐based cases for this robotic system utilising the X‐atlas® Platform (Zimmer Biomet), which relies on standardised long‐leg anteroposterior and lateral radiographs to create 3D models and is similar to MRI‐reconstructed bone models [10]. This technology could be used in future studies to minimise postoperative radiographic measurement errors.

## CONCLUSIONS

This robotic platform performs highly accurate and precise intraoperative bony resections and results in acceptable accuracy using either measured resection or gap balancing with various alignment philosophies. Few outliers (>3° from planned) were identified when coronal and sagittal parameters were measured on postoperative radiographs. Further research is needed to determine where these errors occur and if intra‐operative anatomical landmark selection can be improved.

## AUTHOR CONTRIBUTIONS

**Faseeh Zaidi**: Study conception and design; data acquisition; formal analysis; methodology; writing—original draft; writing—review and editing; funding acquisition. **Craig M. Goplen**: Data acquisition; methodology; writing—original draft; writing—review and editing. **Connor Fitz‐Gerald**: Data acquisition; writing—review and editing. **Scott M. Bolam**: Study conception and design; formal analysis; methodology; writing—review and editing. **Michael Hanlon**: Writing—review and editing. **Jacob T. Munro**: Writing—review and editing. **Andrew P. Monk**: Study conception and design; methodology; writing—review and editing; **Funding acquisition**: Supervision. All authors have read and agreed to the published version of the manuscript. All authors have approved the manuscript and agree with its submission.

## CONFLICT OF INTEREST STATEMENT

Andrew Paul Monk receives research support from and remains a consultant to Zimmer Biomet. The remaining authors declare no conflict of interest.

## ETHICS STATEMENT

Ethical approval was obtained from the local and institutional ethics committee, Auckland Health Research Ethics Committee (AHREC: 000072). Informed consent was obtained from all individual participants included in the study.

## Supporting information

Supporting information.

## Figures and Tables

**Figure A1:**
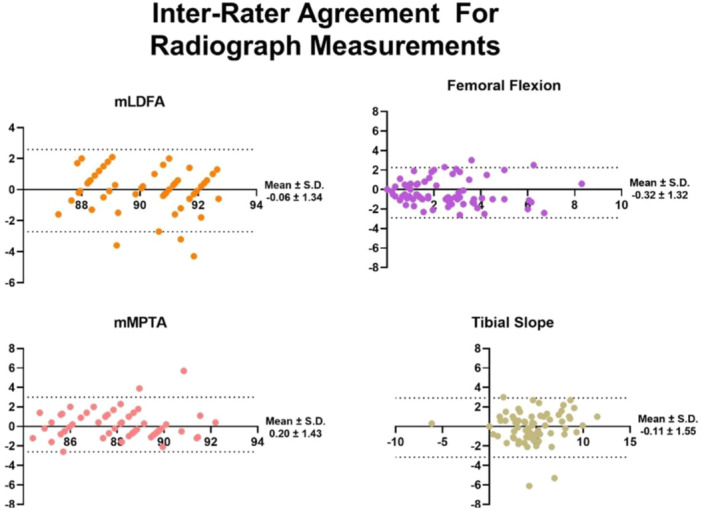
Inter‐rater agreement for radiograph measurements using Bland–Altman plots. mLDFA, mechanical lateral distal femoral angle; mMPTA, mechanical medial proximal tibial angle.

## Data Availability

The data sets used and/or analysed during the current study are available from the corresponding author upon reasonable request.

## APPENDIX A

Figure A1

## References

[1] An, H.M. , Wen, J.X. , Gu, W. , Chen, J.Y. , Chai, W. & Li, R. (2024) Discrepancies in sagittal alignment of the lower extremity among different brands of robotic total knee arthroplasty systems. The Journal of Arthroplasty . In press. Available from: 10.1016/j.arth.2024.03.029 38508345

[2] Bolam, S.M. , Tay, M.L. , Zaidi, F. , Sidaginamale, R.P. , Hanlon, M. , Munro, J.T. et al. (2022) Introduction of ROSA robotic‐arm system for total knee arthroplasty is associated with a minimal learning curve for operative time. Journal of Experimental Orthopaedics, 9, 86. Available from: 10.1186/s40634-022-00524-5 36042122 PMC9427173

[3] Cantivalli, A. , Cottino, U. , Bonasia, D.E. , Rosso, F. & Rossi, R. (2023) Robotic systems in knee surgery: current concepts and future. Prosthesis, 5(4), 1257–1274. Available from: 10.3390/prosthesis5040086

[4] Charette, R.S. , Sarpong, N.O. , Weiner, T.R. , Shah, R.P. & Cooper, H.J. (2022) Registration of bony landmarks and soft tissue laxity during robotic total knee arthroplasty is highly reproducible. Surgical Technology Online, 41, 1633. Available from: 10.52198/22.STI.41.OS1633 36108169

[5] Garfjeld Roberts, P. , Glasbey, J.C. , Abram, S. , Osei‐Bordom, D. , Bach, S.P. & Beard, D.J. (2020) Research quality and transparency, outcome measurement and evidence for safety and effectiveness in robot‐assisted surgery: systematic review. BJS Open, 4, 1084–1099. Available from: 10.1002/bjs5.50352 33052029 PMC7709372

[6] Hampp, E. , Chughtai, M. , Scholl, L. , Sodhi, N. , Bhowmik‐Stoker, M. , Jacofsky, D. et al. (2019) Robotic‐arm assisted total knee arthroplasty demonstrated greater accuracy and precision to plan compared with manual techniques. The Journal of Knee Surgery, 32, 239–250. Available from: 10.1055/s-0038-1641729 29715696

[7] Hasegawa, M. , Tone, S. , Naito, Y. & Sudo, A. (2024) Comparison of accuracy and early outcomes in robotic total knee arthroplasty using NAVIO and ROSA. Scientific Reports, 14, 3192. Available from: 10.1038/s41598-024-53789-4 38326363 PMC10850152

[8] Hasegawa, M. , Tone, S. , Naito, Y. & Sudo, A. (2022) Two‐ and three‐dimensional measurements following robotic‐assisted total knee arthroplasty. The International Journal of Medical Robotics and Computer Assisted Surgery, 18, e2455. Available from: 10.1002/rcs.2455 35993231

[9] Mancino, F. , Rossi, S.M.P. , Sangaletti, R. , Lucenti, L. , Terragnoli, F. & Benazzo, F. (2023) A new robotically assisted technique can improve outcomes of total knee arthroplasty comparing to an imageless navigation system. Archives of Orthopaedic and Trauma Surgery, 143, 2701–2711. Available from: 10.1007/s00402-022-04560-9 35913518

[10] Massé, V. & Ghate, R.S. (2021) Using standard X‐ray images to create 3D digital bone models and patient‐matched guides for aiding implant positioning and sizing in total knee arthroplasty. Computer Assisted Surgery, 26, 31–40. Available from: 10.1080/24699322.2021.1894239 33721547

[11] Meneghini, R.M. , Mont, M.A. , Backstein, D.B. , Bourne, R.B. , Dennis, D.A. & Scuderi, G.R. (2015) Development of a modern knee society radiographic evaluation system and methodology for total knee arthroplasty. The Journal of Arthroplasty, 30, 2311–2314. Available from: 10.1016/j.arth.2015.05.049 26122112

[12] Pailhé, R. (2021) Total knee arthroplasty: latest robotics implantation techniques. Orthopaedics & Traumatology: Surgery & Research, 107, 102780. Available from: 10.1016/j.otsr.2020.102780 33333275

[13] Parratte, S. , Price, A.J. , Jeys, L.M. , Jackson, W.F. & Clarke, H.D. (2019) Accuracy of a new robotically assisted technique for total knee arthroplasty: a cadaveric study. The Journal of Arthroplasty, 34, 2799–2803. Available from: 10.1016/j.arth.2019.06.040 31301912

[14] Parsley, B.S. (2018) Robotics in orthopedics: a brave new world. The Journal of Arthroplasty, 33, 2355–2357. Available from: 10.1016/j.arth.2018.02.032 29605151

[15] Radtke, K. , Becher, C. , Noll, Y. & Ostermeier, S. (2010) Effect of limb rotation on radiographic alignment in total knee arthroplasties. Archives of Orthopaedic and Trauma Surgery, 130, 451–457. Available from: 10.1007/s00402-009-0999-1 19898854

[16] Rossi, S.M.P. , Sangaletti, R. , Perticarini, L. , Terragnoli, F. & Benazzo, F. (2023) High accuracy of a new robotically assisted technique for total knee arthroplasty: an in vivo study. Knee Surgery, Sports Traumatology, Arthroscopy, 31, 1153–1161. Available from: 10.1007/s00167-021-06800-8 PMC8723813 34981162

[17] Schrednitzki, D. , Horn, C.E. , Lampe, U.A. & Halder, A.M. (2023) Imageless robotic‐assisted total knee arthroplasty is accurate in vivo: a retrospective study to measure the postoperative bone resection and alignment. Archives of Orthopaedic and Trauma Surgery, 143, 3471–3479. Available from: 10.1007/s00402-022-04648-2 36269397

[18] Seidenstein, A. , Birmingham, M. , Foran, J. & Ogden, S. (2021) Better accuracy and reproducibility of a new robotically‐assisted system for total knee arthroplasty compared to conventional instrumentation: a cadaveric study. Knee Surgery, Sports Traumatology, Arthroscopy, 29, 859–866. Available from: 10.1007/s00167-020-06038-w 32448945

[19] Shin, C. , Crovetti, C. , Huo, E. & Lionberger, D. (2022) Unsatisfactory accuracy of recent robotic assisting system ROSA for total knee arthroplasty. Journal of Experimental Orthopaedics, 9, 82. Available from: 10.1186/s40634-022-00522-7 35984537 PMC9391541

[20] Siddiqi, A. , Horan, T. , Molloy, R.M. , Bloomfield, M.R. , Patel, P.D. & Piuzzi, N.S. (2021) A clinical review of robotic navigation in total knee arthroplasty: historical systems to modern design. EFORT Open Reviews, 6, 252–269. Available from: 10.1302/2058-5241.6.200071 34040803 PMC8142596

