# Supplementary material for: High in‐vivo accuracy of a novel robotic‐arm‐assisted system for total knee arthroplasty
Source: Knee Surg Sports Traumatol Arthrosc. 2024 May 20;33(1):229–38. doi: 10.1002/ksa.12272 (PMC11716351; doi:10.1002/ksa.12272)
Supplement: Supplementary file 1 — Supporting information. [file KSA-33-229-s001.docx]

**Supplementary Data:**

**Table S1.** Comparison between the final intra-operative planned resection angle, ROSA-validated, and post-operative radiographic coronal and sagittal alignments.

|  | Planned | Validated | Radiographic |  |
| --- | --- | --- | --- | --- |
| Angle | **Mean ± SD** | **Mean ± SD** | **Mean ± SD** | **p-value** |
| mLDFA | 90.13 ± 0.85 | 89.81 ± 0.98 | 90.21 ± 1.56 | 0.17 |
| mMPTA | 88.86 ± 1.33 | 88.76 ± 1.66 | 88.63 ± 1.66 | 0.12 |
| Femoral Flexion | 3.01 ± 0.16 | 2.74 ± 0.79 | 2.84 ± 1.19 | 0.74 |
| Tibial Slope | 6.84 ± 0.73 | 6.77 ± 0.96 | 7.30 ± 1.38 | 0.56 |

SD = standard deviation; mLDFA = mechanical lateral distal femoral angle; mMPTA = mechanical medial proximal tibial angle
